# Glyceraldehyde-3-Phosphate Dehydrogenase Interacts with Proapoptotic Kinase Mst1 to Promote Cardiomyocyte Apoptosis

**DOI:** 10.1371/journal.pone.0058697

**Published:** 2013-03-20

**Authors:** Bei You, Shengdong Huang, Qing Qin, Bing Yi, Yang Yuan, Zhiyun Xu, Jianxin Sun

**Affiliations:** 1 Institute of Cardiothoracic Surgery, Changhai Hospital, Second Military Medical University, Shanghai, China; 2 Center for Translational Medicine, Department of Medicine, Thomas Jefferson University, Philadelphia, Pennsylvania, United States of America; Virginia Commonwealth University, United States of America

## Abstract

Mammalian sterile 20-like kinase 1 (Mst1) is a critical component of the Hippo signaling pathway, which regulates a variety of biological processes ranging from cell contact inhibition, organ size control, apoptosis and tumor suppression in mammals. Mst1 plays essential roles in the heart disease since its activation causes cardiomyocyte apoptosis and dilated cardiomyopathy. However, the mechanism underlying Mst1 activation in the heart remains unknown. In a yeast two-hybrid screen of a human heart cDNA library with Mst1 as bait, glyceraldehyde-3-phosphate dehydrogenase (GAPDH) was identified as an Mst1-interacting protein. The interaction of GAPDH with Mst1 was confirmed by co-immunoprecipitation in both co-transfected HEK293 cells and mouse heart homogenates, in which GAPDH interacted with the kinase domain of Mst1, whereas the C-terminal catalytic domain of GAPDH mediated its interaction with Mst1. Moreover, interaction of Mst1 with GAPDH caused a robust phosphorylation of GAPDH and markedly increased the Mst1 activity in cells. Chelerythrine, a potent inducer of apoptosis, substantially increased the nuclear translocation and interaction of GAPDH and Mst1 in cardiomyocytes. Overexpression of GAPDH significantly augmented the Mst1 mediated apoptosis, whereas knockdown of GAPDH markedly attenuated the Mst1 activation and cardiomyocyte apoptosis in response to either chelerythrine or hypoxia/reoxygenation. These findings reveal a novel function of GAPDH in Mst1 activation and cardiomyocyte apoptosis and suggest that disruption of GAPDH interaction with Mst1 may prevent apoptosis related heart diseases such as heart failure and ischemic heart disease.

## Introduction

Mammalian sterile 20—like kinase 1 (Mst1) is an ubiquitously expressed serine/threonine kinase with a similarity to the Hippo kinase from Drosophila and it is a critical component of the Hippo signaling pathway, which regulates a variety of biological processes ranging from cell contact inhibition, cell growth, organ size control, apoptosis and tumor suppression in mammals [1], [2]. Human Mst1 has two caspase cleavage sites located between the catalytic and regulatory domains, which mediate the cleavage of the autoinhibitory domain [3], [4]. Intact Mst1 is localized predominantly in the cytoplasm, however, in response to a variety of apoptotic stimuli, Mst1 is cleaved by caspases to produce a 34–36-kDa N-terminal constitutively active fragment and this cleavage markedly increases Mst1 kinase activity and translocates the cleaved Mst1 to the nucleus where it phosphorylates histone H2B on Ser14, resulting in chromatin condensation, DNA fragmentation, and, ultimately, cell apoptosis [4]–[6]. In addition to Histone H2B, several Mst1 substrates, including FOXO [7]–[9], LATS1/2 [10], [11], JNK [12] and cardiac troponin I [13], have been recently identified. For instance, MST1 has been shown to phosphorylate FOXO and promote FOXO nuclear translocation, thereby inducing apoptosis in neuronal cells [7], [8].

Regulation of Mst1 appears to occur mainly at posttranslational levels. In addition to its activation by proteolytic cleavage, Mst1 autophosphorylation has been proposed to contribute to the kinase activation [14]. Several phosphorylation sites have been identified in Mst1, namely Thr^175^, Thr^177^, Thr^183^, Thr^187^, Ser^327^ and Thr^387^, of which, Thr^183^ and Thr^187^ appear to be essential for kinase activation [14]–[16]. In addition, protein-protein interactions have also been shown to play critical roles in the regulation of Mst1 activity. Thus far, several proteins including Ras association domain family protein (Rassf) [16]–[18], hWW45 [17], [19], PHLPP1 [20], and Heat Shock Protein 70 (Hsp70) [21], have been identified to interact with Mst1 and regulate Mst1 activation. For instance, RASSF family of tumor suppressors have been shown to interact with and stabilize Mst1, thereby preventing Mst1 for the degradation and inhibiting tumor growth [18], [22]. In contrast, our recent results demonstrated that Hsp70 decreases Mst1 activity through promoting Mst1 degradation via a CHIP dependent pathway, thereby preventing cancer cells from cisplatin induced apoptosis [21].

Recently, the physiological role of Mst1 in the cardiovascular system has begun to be explored. In cardiomyocytes, Mst1 is activated by pathological stimuli, such as hypoxia/reoxygenation in vitro and ischemia/reperfusion in vivo [23]. Cardiac-specific over-expression of Mst1 has been shown to cause dilated cardiomyopathy in mice [23]. Inhibition of endogenous Mst1 prevents apoptosis of cardiomyocytes and cardiac dysfunction after myocardial infarction without producing cardiac hypertrophy [23], [24]. Recently, we identified Mst1 as a novel kinase that mediates cTnI phosphorylation and plays a critical role in the modulation of myofilament function in the heart [13]. However, despite these important functions, little is relatively known about the mechanisms underlying the regulation of the Mst1 activation in the heart. In an attempt to identify novel cardiac proteins that may associate with Mst1 and regulate Mst1 activation in the heart, we performed yeast two-hybrid screen of a human heart cDNA library using the dominant negative form of Mst1 (K59R) as bait and subsequently, we identify glyceraldehyde-3-phosphate dehydrogenase (GAPDH) as a novel Mst1-interacting protein that positively regulates Mst1 activation and cell apoptosis in cardiomyocytes.

## Materials and Methods

### Cell culture

HEK293T and Ad293 cells were cultured in Dulbecco's Modified Eagle's Medium (DMEM) (Invitrogen) supplemented with 10% Fetal Bovine Serum (FBS) (Gibco) and were maintained at 37°C with 5% CO2. Cells were transiently transfected with FuGENE 6 (Roche Applied Science) using the manufacturer's protocol.

### Yeast Two-Hybrid library screening and interaction assays

To identify novel proteins that interact with Mst1, we screened a human heart cDNA library using human dominant-negative Mst1 (K59R) as bait and the MATCHMAKER GAL4 yeast 2-hybrid system 3 (Clontech Laboratories Inc) as previously described [13], [21]. The screening was performed in AH109 yeast cells according to the protocol provided by the Clontech Matchmaker Two-Hybrid System. Positive colonies were subject to multiple rounds of additional selection in the appropriate medium and β-galactosidase (β-gal) filter assays to verify specificity.

### Primary culture of neonatal rat ventricular myocytes (NRVMs) and hypoxia/reoxygenation injury

We obtained ventricles from 1-day-old Sprague-Dawley rats and isolated cardiac myocytes by digestion with trypsin-EDTA and type 2 collagenase as previously described [25]. Neonatal rats were obtained from Charles River Laboratories North Wilmington, Mass. This study was reviewed and approved by the Institutional Animal Care and Use Committee at Thomas Jefferson University. Hypoxia was achieved by placing the cells in a hypoxia chamber filled with 5% CO_2_ and 95% N_2_ at 37°C for 12 hours. Following hypoxia exposure, cells were reoxygenated by placing cells in the normoxic culture medium for 24 hours 26]

### Coimmunoprecipitation of Mst1 and GAPDH in HEK293T cells and cardiomyocytes

HEK293T cells were transiently transfected with Mst1 and GAPDH cDNAs using FuGENE 6 as described by the manufacturer (Roche Applied Science). Both HEK293T cells and cardiomyocytes were lysed in the buffer containing 1% Nonidet P-40, 150 mmol/L NaCl, 50 mmol/L Tris (pH 8), 100 µmol/L EDTA, and protease inhibitors. Coimmunoprecipitation of Mst1 and GAPDH was performed essentially as described [25]. The following antibodies were used for detection and immunoprecipitation: rabbit polyclonal c-Myc (Invitrogen), mouse monoclonal FLAG M2 (Sigma), mouse monoclonal GAPDH (Abcam), rabbit polyclonal GAPDH (Abcam), anti-Mst1 monoclonal antibody (BD Transduction Laboratories) and rabbit polyclonal Mst1 (Abgent). Secondary antibodies were peroxidase-conjugated donkey anti-rabbit or anti-mouse (Jackson ImmunoResearch). Detection of the peroxidase was performed with ECL reagents.

### Immunofluorescence staining

Freshly isolated neonatal rat cardiomyocytes were fixed and sequentially incubated with primary antibodies and appropriate fluorescent-labeled secondary antibodies. Images were visualized using an Olympus IX70 epifluorescence microscope as previously described [25].

### In Vitro GAPDH phosphorylation assay

To phosphorylate GAPDH *in vitro*, recombinant GAPDH (Abcam) was incubated with recombinant active Mst1 (Upstate) and [γ-^32^P]-ATP (PerkinElmer) in 1× kinase assay buffer (40 mM HEPES-NaOH pH 7.4, 20 mM MgCl_2_, 1 mM DTT, 1 mM ATP, 1 mM Na_3_VO_4_, 50 mM NaF, and complete protease inhibitor (Roche) at 30 °C for indicated time points. The reaction was terminated by adding 1/2 volumes of 3× Laemmli sample buffer and incubated at 95 °C for 5 min, then examined by 15% SDS-PAGE and a autoradiography.

### Mst1 activity assay

Recombinant active Mst1 (Upstate) or immunoprecipitated Mst1 from HEK293T transfected with Myc-Mst1 cDNA in combination with either empty vector or Flag-GAPDH was incubated for 20 min at 30°C with 4 µg Myelin basic protein (MBP) (Upstate) in 25 µL kinase buffer 40 mmol/L HEPES-NaOH (pH 7.4), 20 mmol/L MgCl_2_, 1 mmol/L DTT, and 1 µCi [γ-^32^P]-ATP. Reactions were terminated by the addition of 2× SDS sample buffer, and then loaded to 15% SDS-PAGE and subjected to autoradiography. For Mst1 kinase assay in cardiomyocytes, cell homogenates (400 µg) were immunoprecipitated using anti-Mst1 antibody (BD Transduction Laboratories), and then incubated with 2 µg Histone H2B (Sigma) in 25 µL kinase assay buffer for 20 min at 30°C. Samples were subjected to SDS-PAGE and phosphorylation of histone H2B was detected by immunoblotting with anti-phospho-Histone H2B (Ser14) antibody (Millipore).

### Small interfering RNA of GAPDH

Predesigned GAPDH siRNA and control siRNA were purchased from Ambion. Cardiomyocytes were transfected with Lipofectamine™ RNAiMAX Transfection Reagent (Invitrogen) with target-specific siRNA (20 nmol/L) and control siRNA (20 nmol/L) in serum-free medium according to the recommendations of the manufacturer.

### Construction of adenoviruses

Adenoviruses harboring wild-type Mst1 (Ad-Mst1), dominant-negative Mst1 (Ad-DNMST), and GAPDH were made using AdMax (Microbix) as previously described [21]. Briefly, pBHGloxΔE1,3XCre, including the ΔE1 adenoviral genome, was cotransfected with the pDC shuttle vector containing the gene of interest into AD-293 cells (Stratagene, La Jolla, CA) using FuGene 6 Transfection Reagent (Roche, Indianapolis, IN). AD-293 cell line is a derivative of the commonly used HEK293 cell line, with improved cell adherence and plaque formation properties. The viruses were propagated in AD-293 cells and purified using CsCl_2_ banding, followed by dialysis against 20 mmol/L Tris-buffered saline with 10% glycerol. Titering was performed on Ad293 cells using Adeno-X Rapid Titer kit (Clontech) according to the instructions of the manufacturer.

### Induction of cardiac hypertrophy by chronic infusion of isoproterenol (ISO)

C57BL/6 mice (20–25 g) were anesthetized with a mixture of ketamine (50 mg/kg) and xylazine (2.5 mg/kg) intraperitoneally. The mini-osmotic pumps (Alza corporation, Pa, Alto, CA) set to dispense 0.5 µl/hr for 14 days were filled with PBS or ISO (60 mg/kg/day) and pumps were implanted subcutaneously in the back through a sub-scapular incision, which was then closed using 4.0 silk suture (Ethicon). Mice were followed by echocardiogram prior to pump implantation and then weekly until the end of the 2 week period when mice developed cardiac hypertrophy. This study was reviewed and approved by the Institutional Animal Care and Use Committee at Thomas Jefferson University. Tissue homogenates from normal heart and hypertrophied heart were then subjected to immunoprecipitation by using anti-Mst1 antibody as previously described [25]

### Detection of apoptosis

Histone-associated DNA fragments were quantitated by the Cell Death Detection ELISA kit (Roche) [25]. Briefly, the cytoplasmic fractions were added to the 96-well ELISA plates precoated with the antihistone monoclonal antibody and incubated for 90 min at room temperature. After washing, bound nucleosomes were detected by the addition of anti–DNA-peroxidase monoclonal antibody and reacted for 60 min at room temperature. After the addition of substrate, absorbance was read with an ELISA reader at 405 nm. Cardiomyocyte apoptosis measured by using Terminal Transferase-mediated dUTP nick-end labeling (TUNEL) staining was performed using the In Situ Cell Death Detection kit (Roche).

### Statistical analyses

Data are expressed as means ± SEM. The statistical significance of differences was assessed by Student's *t* tests or ANOVA, as appropriate; a value of *P*<0.05 was considered statistically significant.

## Results

### Interaction of GAPDH with Mst1

In an effort to identify novel Mst1 binding proteins, a yeast 2-hybrid screening was performed with human dominant negative Mst1 (DN-Mst1) as bait in conjunction with a human heart cDNA library. After screening 2.4×10^6^ clones, we identified 4 positive cDNAs encoding the C-terminal domain (aa 164–335) of GAPDH, suggesting that the C-terminal region of GAPDH may be sufficient to interact with Mst1 in yeast.

**Figure 1:**
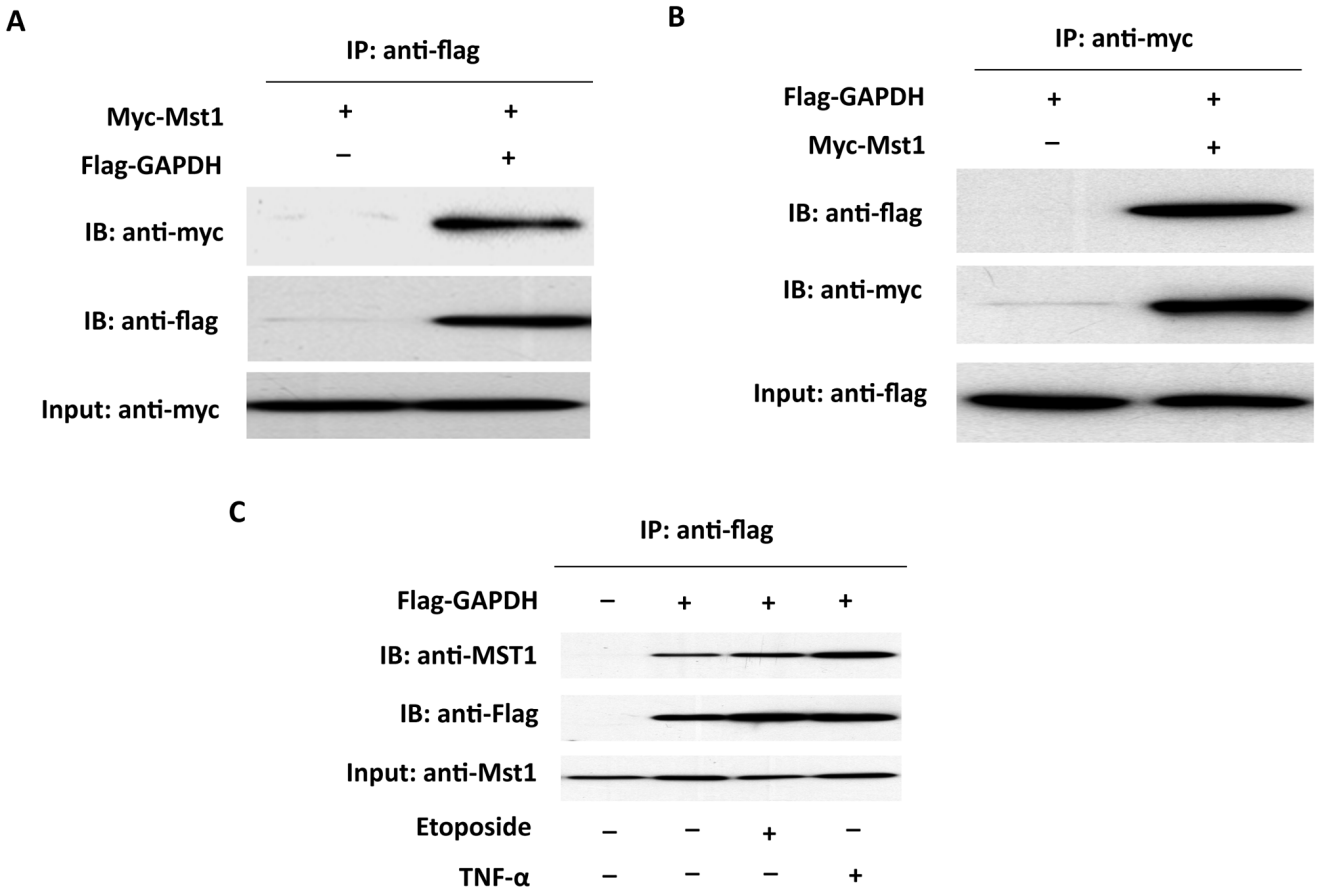
Physical interaction between Mst1 and GAPDH by immunoprecipitation analysis. A, Myc-Mst1 expression vector in combination of either empty vector or pFlag-GAPDH were co-transfected into HEK293 cells. Extracted proteins were precipitated by anti-FLAG antibody and then separated by 12% SDS-PAGE. The transferred membrane was immunoblotted with either HRP conjugated anti-Myc or HRP conjugated anti-FLAG antibody. B, Flag-GAPDH expression vector in combination of either empty vector, pMT2-Myc-Mst1 were co-transfected into HEK293 cells. Extracted proteins were precipitated by anti-Myc antibody and then separated by 12% SDS-PAGE. The transferred membrane was immunoblotted with either HRP conjugated anti-Myc or HRP conjugated anti-FLAG antibody. C, HEK293 cells were transfected with Flag-Mst1. 48 hr after transfection, cells were then treated with etoposide (100 M) or TNF- (20 ng/ml) for 6 hrs. Extracted proteins were precipitated by anti-Flag antibody and then separated by 12% SDS-PAGE. The transferred membrane was immunoblotted with either anti-Mst1 or anti-Flag antibodies.

To determine whether the interaction between Mst1 and GAPDH occurs in mammalian cells, coimmunoprecipitation experiments were performed in HEK293T cells transfected with GAPDH and Mst1 expression vectors. Immunoprecipitation of FLAG-tagged GAPDH led to coimmunoprecipitation of Myc-tagged Mst1 when both proteins were cotransfected (Figure 1A). As a control, the anti-FLAG antibody did not immunoprecipitate Myc-tagged Mst1 in the absence of FLAG-GAPDH. Similarly, immunoprecipitation of Myc-tagged Mst1 resulted in coimmunoprecipitation of FLAG-tagged GAPDH, whereas the anti-Myc antibody did not immunoprecipitate FLAG-GAPDH in the absence of Myc-Mst1 (Figure 1B). Interestingly, the interaction of GAPDH with Mst1 was further increased by treatment of the cells with the apoptotic agent etoposide (100 M) or TNF- (20 ng/ml) for 6 hrs (Figure 1C). Together, these findings indicate that GAPDH and Mst1 exist in the same complex in mammalian cells both at baseline conditions and during apoptotic stress.

**Figure 2:**
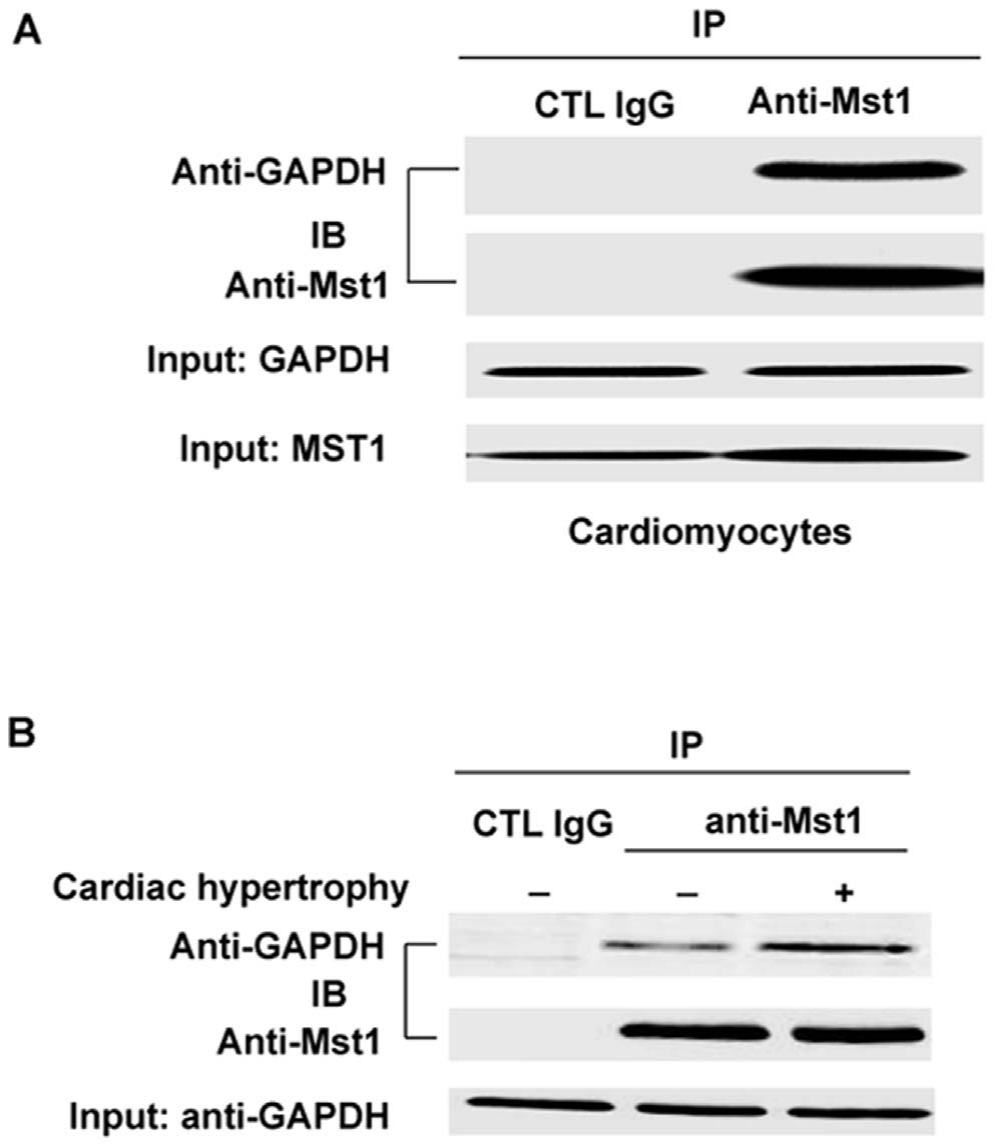
Association of Mst1 with GAPDH in cardiomyocytes. A. Cell lysates obtained from NRVMs were immunoprecipitated with anti-Mst1 antibody and then separated by 12% SDS-PAGE. Transferred membrane was immunoblotted with either anti-GAPDH or Mst1 antibody. B, Tissue homogenates obtained from normal mouse heart and hypertrophic heart were immunoprecipitated with anti-Mst1 antibody and then separated by 12% SDS-PAGE. Transferred membrane was immunoblotted with either anti-GAPDH or Mst1 antibody.

To determine whether there is an endogenous interaction of GAPDH and Mst1 in cardiomyocytes, we performed immunoprecipitation with anti-Mst1 antibody using lysates obtained from neonatal rat ventricular cardiomyocytes (NRVMs). Indeed, GAPDH was co-precipitated with the anti-Mst1 antibody but not with the nonimmune IgG (Figure 2A). The interaction of GAPDH and Mst1 was further examined in the heart. Similarly, GAPDH was only co-immunoprecipitated with the anti-Mst1 antibody, but not with the nonimmune IgG in mouse heart homogenates and this interaction was further increased in the hypertrophic heart (Figure 2B). The results indicate that GAPDH interacts with Mst1 in cardiomyocytes under physiological and pathophysiological conditions.

### Interaction domains of GAPDH and Mst1

**Figure 3:**
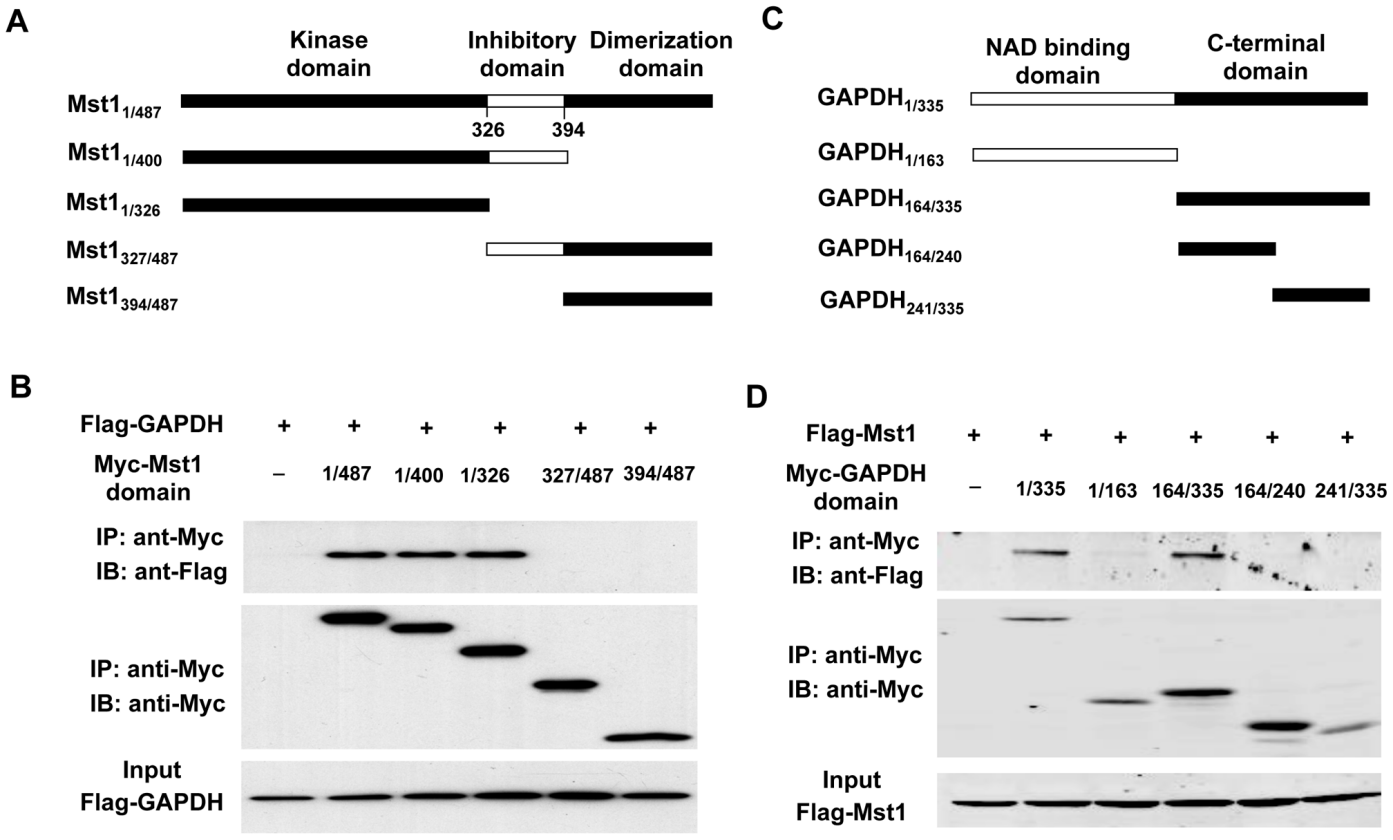
Identification of Mst1 and GAPDH interaction sites. *A,* schematic representation of Mst1 deletion mutants. *B,* flag-GAPDH expression vector in combination of either empty vector or expression vectors of Myc-Mst1 mutants were cotransfected into HEK293T cells. Extracted proteins were precipitated by anti-Myc antibody and then separated by 12% SDS-PAGE. The transferred membrane was immunoblotted with either HRP-conjugated anti-FLAG or HRP-conjugated anti-Myc antibody. Lysates were also immunoblotted with anti-Flag antibody to show the expression levels of Flag-GAPDH in HEK293T cells. *C,* schematic representation of GAPDH deletion mutants. *D,* Flag-Mst1 expression vector in combination of either empty vector or expression vectors of Myc-GAPDH mutants were cotransfected into HEK293T cells. Extracted proteins were precipitated by anti-Myc antibody and then separated by 12% SDS-PAGE. The transferred membrane was immunoblotted with either HRP-conjugated anti-FLAG or HRP-conjugated anti-Myc antibody. Lysates were also immunoblotted with anti-Flag antibody to show the expression of Flag-Mst1 in HEK293 cells.

To further map the interaction domains of GAPDH and Mst1, we performed immunoprecipitation in HEK293 cells cotransfected with different deletion mutants of Mst1 and GAPDH. We first investigated the binding domains of GAPDH in Mst1. Mst1 contains an N-terminal kinase domain (aa 1–325), inhibitory domain (aa 326–294), and a C-terminal dimerization domain (aa 395–487) [3] (Figure 3A). We generated a series of Mst1 deletion mutants subcloned into pCS26MT vector with 6× Myc tag and transfected these mutants into HEK293 cells along with Flag-GAPDH. Lysates from transfected HEK293T cells were immunoprecipitated with anti-myc antibody and analyzed by Western blot analysis using anti-Flag and anti-Myc antibodies. We found that Flag-GAPDH only bound to the N-terminal kinase domain of Mst1 (figure 3B), suggesting that the kinase domain of Mst1 is necessary for interaction with GAPDH.

GAPDH contains two functional domains, namely, the NAD binding domain and the C-terminal catalytic domain [27] (Figure 3C). To map the Mst1-binding domain in GAPDH, Myc-tagged GAPDH mutants were cotransfected into HEK293T cells with Flag-tagged Mst1. Lysates from transfected HEK293T cells were immunoprecipitated with anti-Myc antibody and analyzed by Western blotting analysis using anti-Flag and anti–Myc-antibodies. As shown in Figure 3D, Mst1 interacted only with the entire C-terminal catalytic domain of GAPDH, but not with the NAD binding domain and the deletion mutants derived from the C-terminal catalytic domain. Together, these results further suggest that the C-terminal catalytic domain of GAPDH mediates its interaction with Mst1.

### Phosphorylation of GAPDH by Mst1

**Figure 4:**
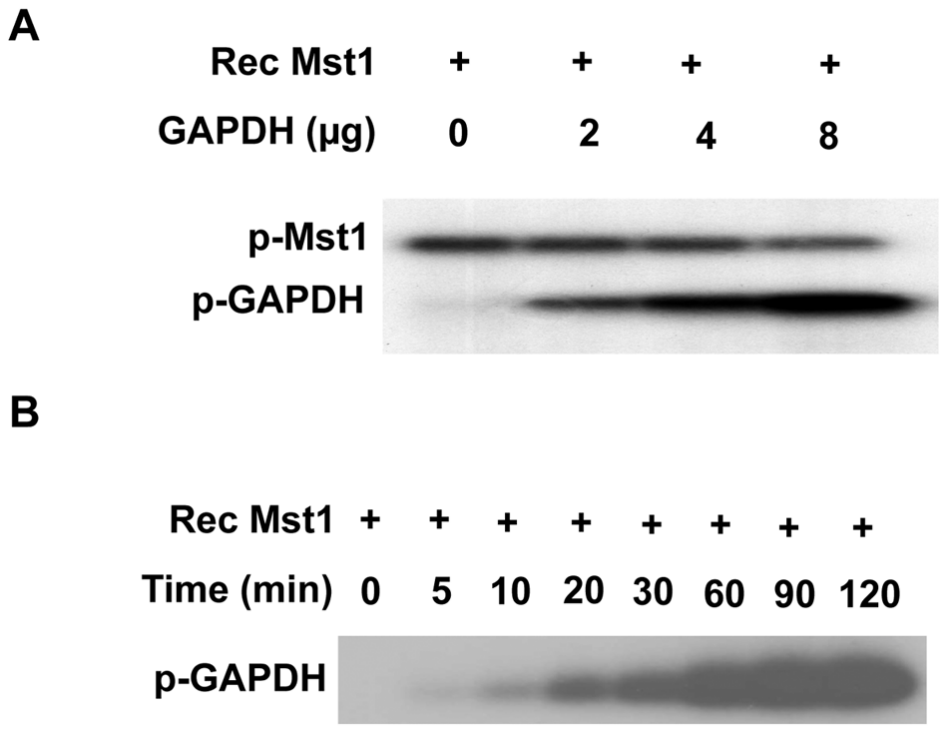
Phosphorylation of GAPDH by Mst1. A, 0.1 µg active Mst1 was incubated with different amounts of recombinant GAPDH for 60 min in the presence of ^32^P-ATP in an *in vitro* phosphorylation assay. B, 0.1 µg active Mst1 was incubated with 4 µg bacterially expressed recombinant GAPDH for different time points in the presence of ^32^P-ATP in an *in vitro* phosphorylation assay. Phosphorylation was detected by autoradiography. The data are representatives of 4 independent experiments.

Since Mst1 is a serine/threonine kinase [1], the interaction of Mst1 with GAPDH prompted us to investigate whether GAPDH is a substrate of Mst1. Therefore purified human GAPDH was incubated with active recombinant Mst1 in the presence of [γ-^32^P]ATP in an in vitro phosphorylation assay. Indeed, incubation of Mst1 with GAPDH induced a robust phosphorylation of GAPDH in a dose dependent manner (Figure 4A). To determine the kinetics of Mst1-mediated phosphorylation of GAPDH, the time course of Mst1 induced GAPDH phosphorylation was determined in vitro. Indeed, Mst1 phosphorylated GAPDH in a time-dependent manner and reached saturation at 90 min (figure 4B). Strikingly, the activity of either recombinant or endogenous GAPDH was not affected by either incubation with recombinant Mst1 or transduction of cardiomyocytes with adenovirus expressing Mst1 (data not shown).

### Activation of Mst1 by GAPDH

**Figure 5:**
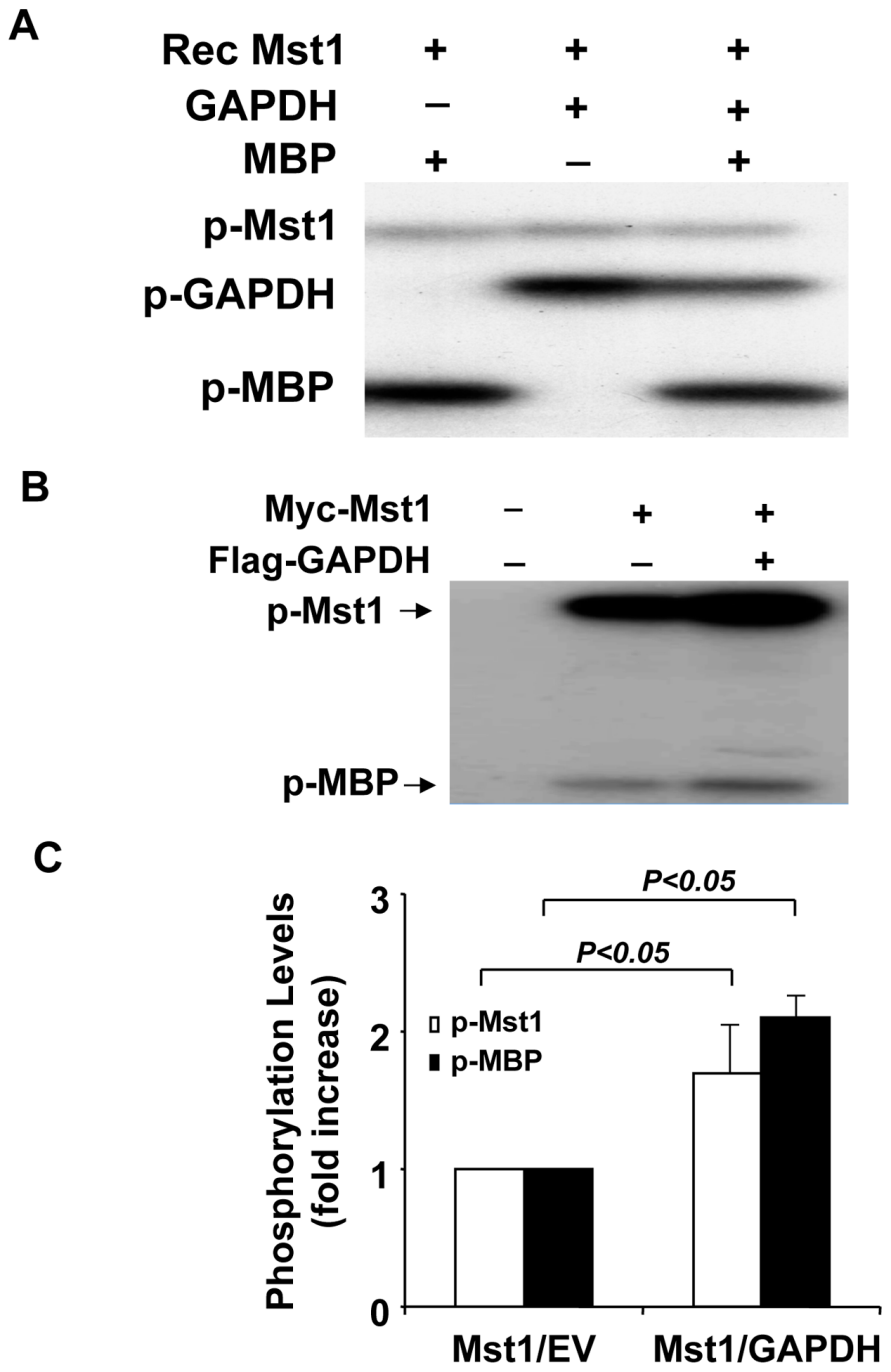
Activation of Mst1 kinase by GAPDH. A, 0.1 µg active Mst1 was incubated with either 4 µg of recombinant GAPDH or MBP for 60 min in the presence of ^32^P-ATP in an *in vitro* phosphorylation assay. Phosphorylation was detected by autoradiography. B, Mst1 immunoprecipitated from HEK293 cells transfected with either Myc-Mst1 or Myc-Mst1 plus Flag-GAPDH expression vectors was incubated with 2 µg MBP for the in vitro kinase assay. Kinase assay was carried out in the presence of ^32^P-ATP for 60 min. Phosphorylation was detected by autoradiography. C, Phosphorylation levels of Mst1 and MBP were quantified by densitometry of autoradiograms. Values are means ± SEM obtained from 3 experiments.

To determine whether the interaction of GAPDH with Mst1 has functional consequences in terms of affecting Mst1 activity, we performed an in vitro kinase assay by using either recombinant Mst1 or Mst1 immunoprecipitated from HEK293 cells transfected with the Myc-Mst1 cDNA together with empty vector or Flag-GAPDH plasmid. As expected, incubation of recombinant Mst1 with its known substrate myelin basic protein (MBP) resulted in a robust phosphorylation of MBP, which was not significantly affected in the presence of GAPDH (Figure 5A). Moreover, the phosphorylation activity of Mst1 on GAPDH was about the same as that on the known substrate MBP (Figure 5A). Interestingly, when Mst1 immunoprecipitated from HEK293T cells was used in the kinase assay, cotransfection of GAPDH substantially increased both the Mst1 autophosphorylation and Mst1 mediated MBP phosphorylation by approximately 2-fold (Figure 5B and 5C). These data suggest that other components present in the Mst1 immunocomplexes, but not in the recombinant Mst1, is required for the activation of Mst1 by GAPDH.

### Nuclear translocation of Mst1 and GAPDH during cardiomyocyte apoptosis

**Figure 6:**
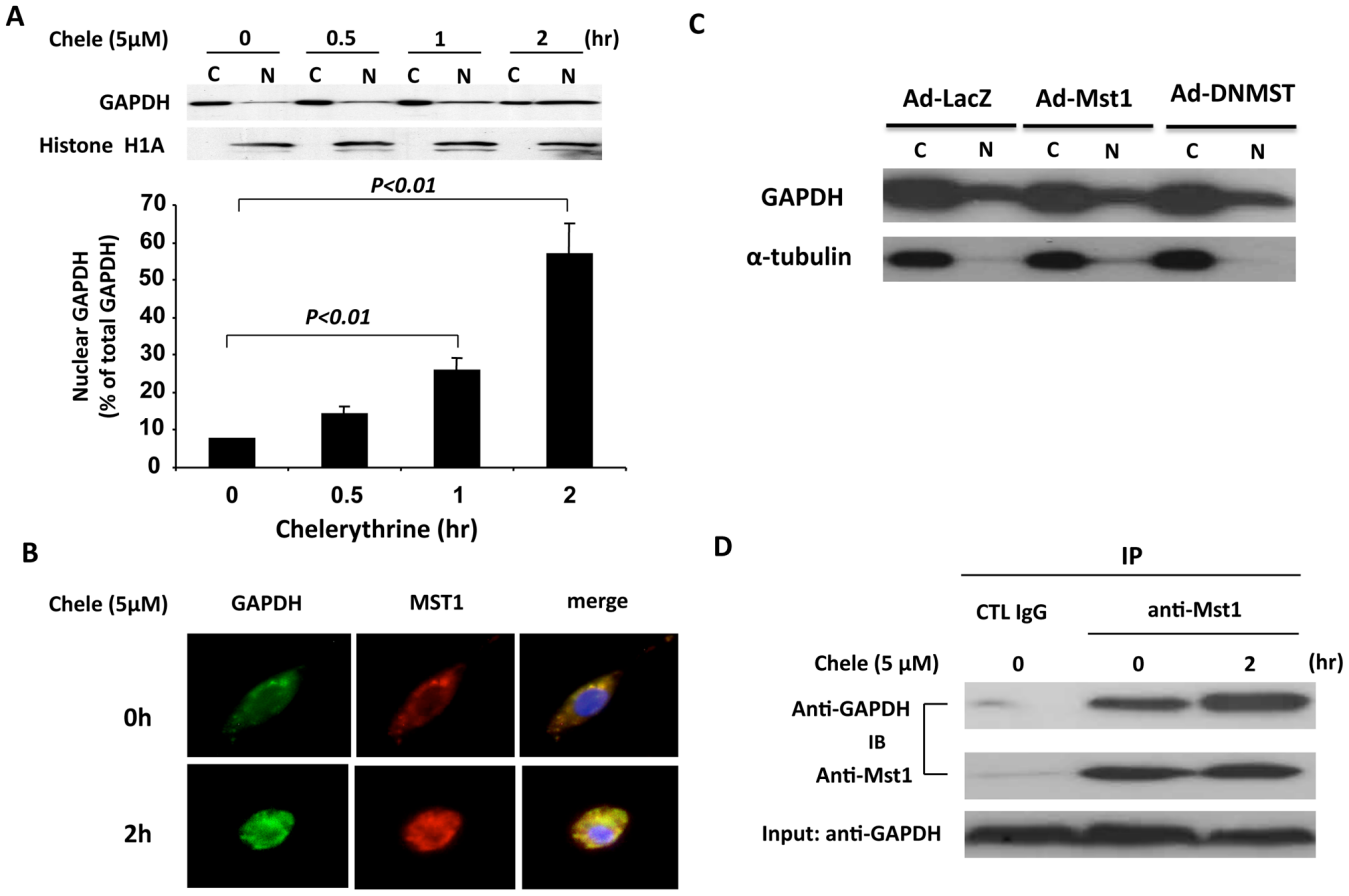
Nuclear translocation of GAPDH and Mst1 during cardiomyocyte apoptosis. A, NRVMs were treated with chelerythrine (5 µM) for different time points as indicated. Cytoplasmic and nuclear fractions were isolated and then subjected to western blot analysis using ant-GAPDH and anti-histone H1A antibodies. The distribution of GAPDH in the cytoplasmic and nuclear fractions was analyzed by densitometric analysis. Values are means ± SEM obtained from 4 experiments. B, Unstimulated NRVMs or NRVMs stimulated with chelerythrine (5 µM) for 2 hours were fixed and stained with anti-GAPDH monoclonal antibody and rabbit polyclonal anti-Mst1 antibody and processed for confocal imaging. The merged images show clear colocalization of these 2 proteins in cytoplasm in ustimulated cells and translocation and colocalization of these 2 proteins in nucleus in response to chelerythrine. C, NRVMs were transduced with either Ad-LacZ or Ad-Mst1 or Ad-DNMST (MOI = 30). 48 hr after transduction, cells were treated with chelerythrine (5 µM) for 1 hour. Cytoplasmic and nuclear fractions were isolated and then subjected to western blot analysis using anti-GAPDH and anti--tubulin antibodies. D, Unstimulated NRVMs or NRVMs stimulated with chelerythrine (5 µM) for 2 hours were lysed and then subjected to immunoprecipitation with either normal IgG or anti-Mst1 antibody. Immunocomplexes were then separated by 15% SDS-PAGE and transferred membrane was immunoblotted with either anti-GAPDH or Mst1 antibody.

GAPDH has been shown to induce cell apoptosis through its nuclear translocation [28]. To determine whether GAPDH is involved in cardiomyocyte apoptosis, neonatal rat cardiomyocytes were treated with chelerythrine, a potent inducer of apoptosis [29]. The distribution of GAPDH between the cytoplamic and nuclear fractions was then determined by western blotting analysis. As shown in Figure 6A, in unstimulated cardiac cells, the majority of GAPDH is located in the cytoplasm. However, treatment of cardiomyocytes with chelerythrine for either 1 hr or 2 hr substantially increased the amount of GAPDH in the nuclear fraction. To further substantiate the role of Mst1 and GAPDH in cardiomyocyte apoptosis, we performed immunofluorescence staining to determine the intracellular localization of GAPDH and Mst1 in cardiomyocytes. As shown in Figure 6B, the majority of GAPDH and Mst1 is colocalized in the cytoplasm in unstimulated cardiac cells. However, chelerythrine treatment led to a marked translocation and co-localization of both GAPDH and Mst1 in the nucleus. The kinase activity of Mst1 is not required for the nuclear translocation of GAPDH, since transduction of cardiomyocytes with adenovirus bearing either wild-type Mst1 (Ad-Mst1, MOI = 30) or dominant negative Mst1 (Ad-DNMST, MOI = 30) had no effect on the nuclear translocation of GAPDH induced by chelerythrine stimulation (Figure 6C). Furthermore, the interaction of GAPDH with Mst1 was further increased in cardiomyocytes in response to chelerythrine treatment, as demonstrated in the co-immunoprecipitation experiment using anti-Mst1 antibody (Figure 6D). Together, these results suggest that translocation of GAPDH and Mst1 into nucleus may play an essential role in Mst1 activation and cardiomyocyte apoptosis.

### GAPDH enhances Mst1 induced cardiomyocyte apoptosis

**Figure 7:**
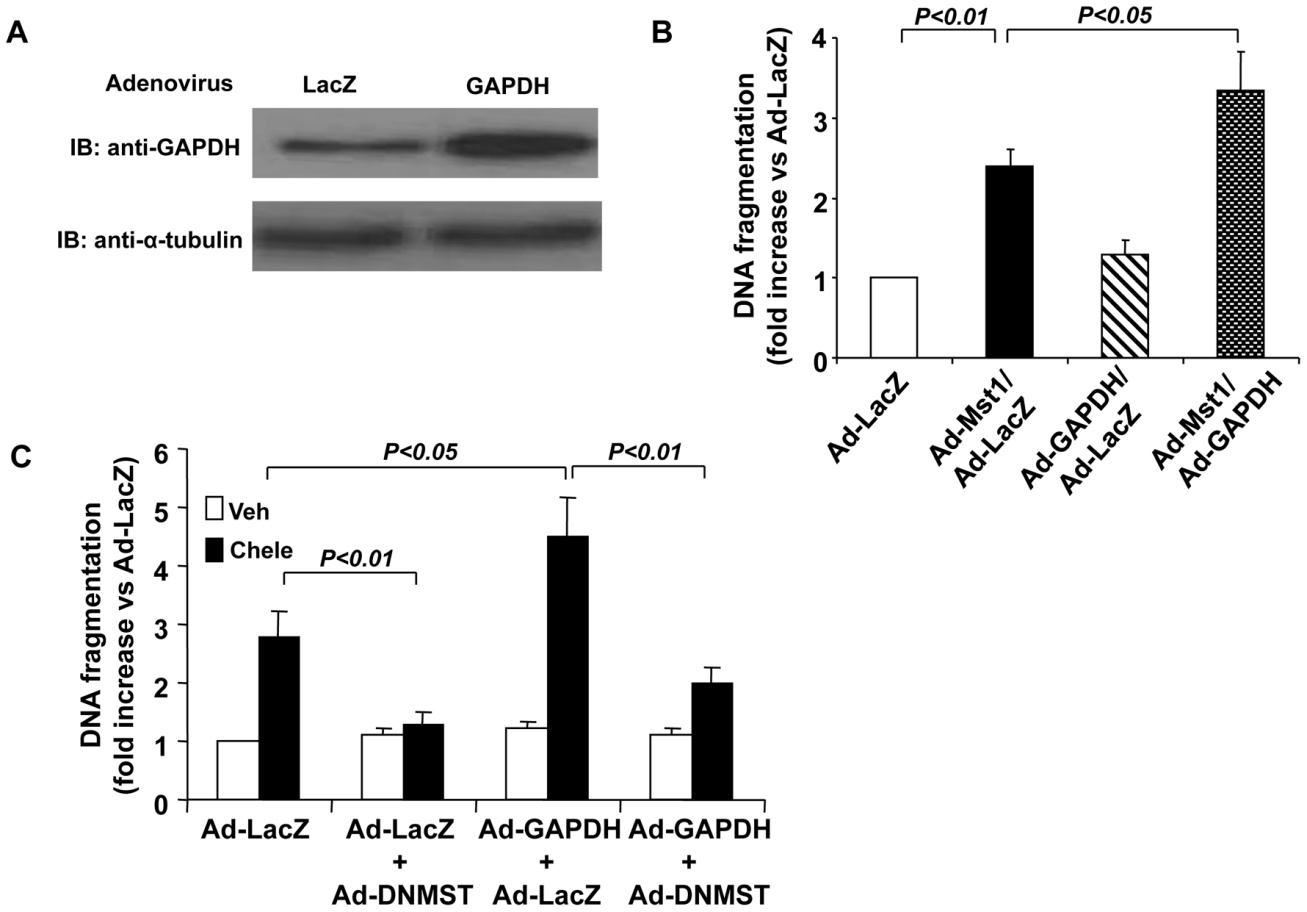
Overexpression of GAPDH enhances Mst1 mediated cardiomyocyte apoptosis. A, NRVMs were transduced with either Ad-LacZ or Ad-GAPDH (MOI = 30). 48 hr after transduction, cell lysates were subjected to western blot analysis to detect the expression of GAPDH. B, NRVMs were transduced with Ad-LacZ or Ad-GAPDH at 30 mois. Twenty-four hours after transduction, myocytes were transduced with Ad-LacZ or Ad-Mst1 at 30 mois. 48 hours after the second transduction, cytoplasmic accumulation of mono- and oligonucleosomes was quantitated by the Cell Death Detection ELISA. Values are means ± SEM obtained from 4 experiments. C, NRVMs were transduced with Ad-LacZ, Ad-GAPDH, or Ad-DNMST with different combinations (total 60 mois). 48 hours after the transduction, the cells were treated with chelerythrine (5 µM) for 2 hours, the cytoplasmic accumulation of mono- and oligonucleosomes was then quantitated by the Cell Death Detection ELISA. Values are means ± SEM obtained from 4 experiments.

Because Mst1 has been characterized to induce cardiomyocyte apoptosis [23], [24], we investigated whether GAPDH can affect cell apoptosis via its stimulatory effect on Mst1 in cardiac myocytes. Indeed, transduction of cardiomyocytes with adenovirus bearing GAPDH (Ad-GAPDH, MOI = 30) resulted in an increased expression of GAPDH (Figure 7A). As expected, transduction of cardiomyocytes with Ad-Mst1 (MOI = 30) significantly induced apoptosis as compared with cells transduced with Ad-lacZ (MOI = 30), as determined by the Cell Death ELISA (Roche) (Figure 7B). However, transduction of cardiomyocytes with Ad-GAPDH (MOI = 30) alone barely affected the basal levels of cell apoptosis, but substantially enhanced the Mst1 induced apoptosis. In addition, overexpression of GAPDH markedly augmented the cardiomyocyte apoptosis in response to chelerythrine stimulation, which was significantly inhibited by overexpression of DN-Mst1 (Figure 7C). These findings suggest that the interaction of GAPDH with Mst1 may be functionally important in terms of regulating Mst1-mediated cardiomyocyte apoptosis.

### Knockdown of GAPDH attenuates Mst1 activation and cardiomyocyte apoptosis

**Figure 8:**
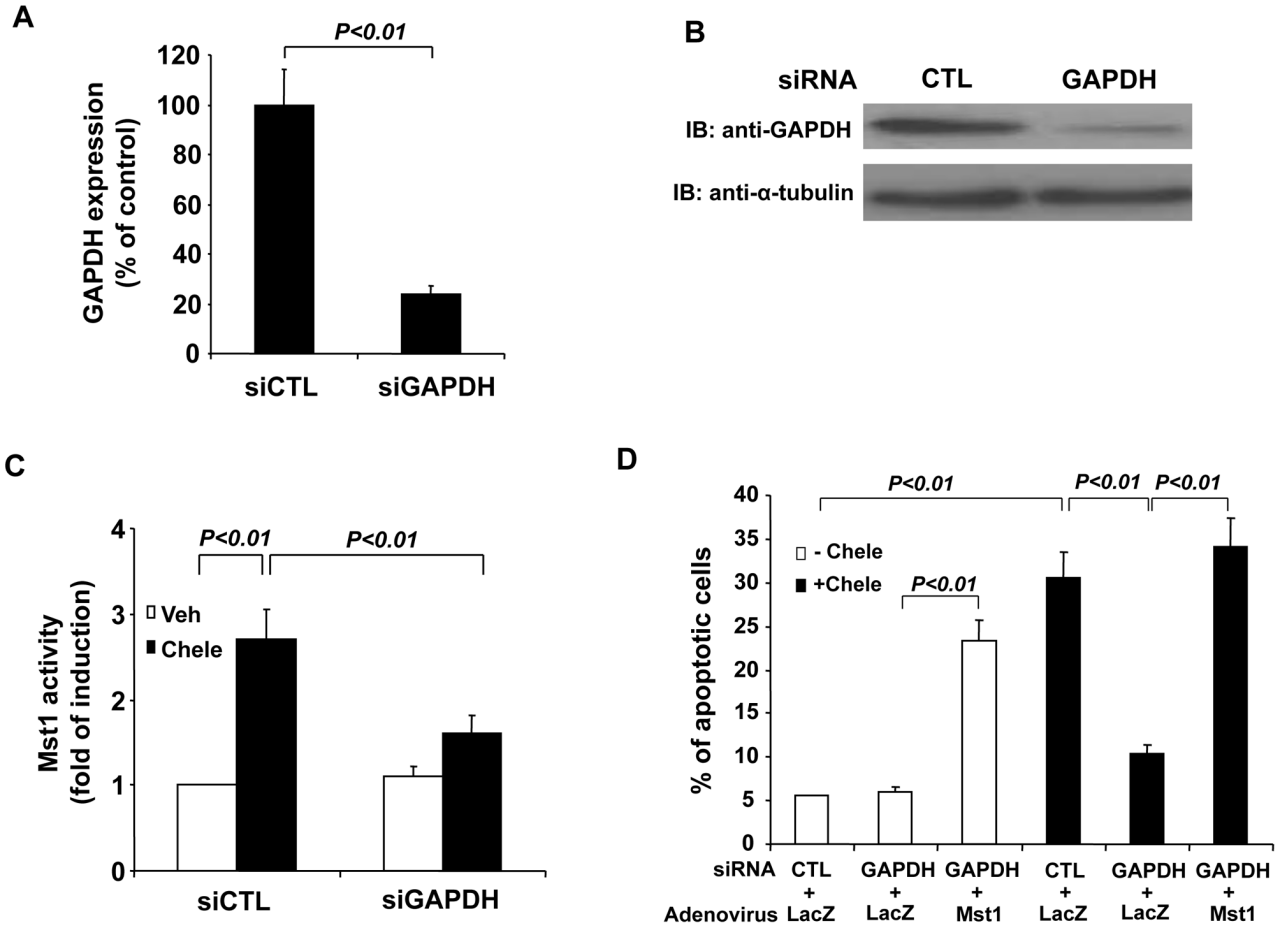
Knockdown of GAPDH attenuates Mst1 activation and cell apoptosis in response to chelerythrine. A, Total RNA extracted from control siRNA (siCTL) and GAPDH siRNA transfected cells was analyzed for the expression of GAPDH in NRCMs by qRT-PCR. B, NRVMs were transfected with either control siRNA or GAPDH siRNA. 72 hours after transfection, cell lysates were then subjected to western blot analysis to detect the expression of GAPDH. C, NRVMs were transfected with either control siRNA or GAPDH siRNA. 72 hours after transfection, cells were treated with chelerythrine (5 µM) for 2 hours. Mst1 was then immunoprecipitated and its activity was determined by an in vitro kinase assay using histone H2B as a substrate. D, NRVMs were transfected with either control siRNA or GAPDH siRNA. 24 hours after siRNA transfection, cells were then transduced with either Ad-LacZ or Ad-Mst1 (MOI = 50). 48 hours after virus transduction, NRVMs were treated with chelerythrine (5 µM) for 2 hours and the cell apoptosis was determined by using the TUNEL staining kit (Roche). Values are means ± SEM obtained from 4 experiments.

**Figure 9:**
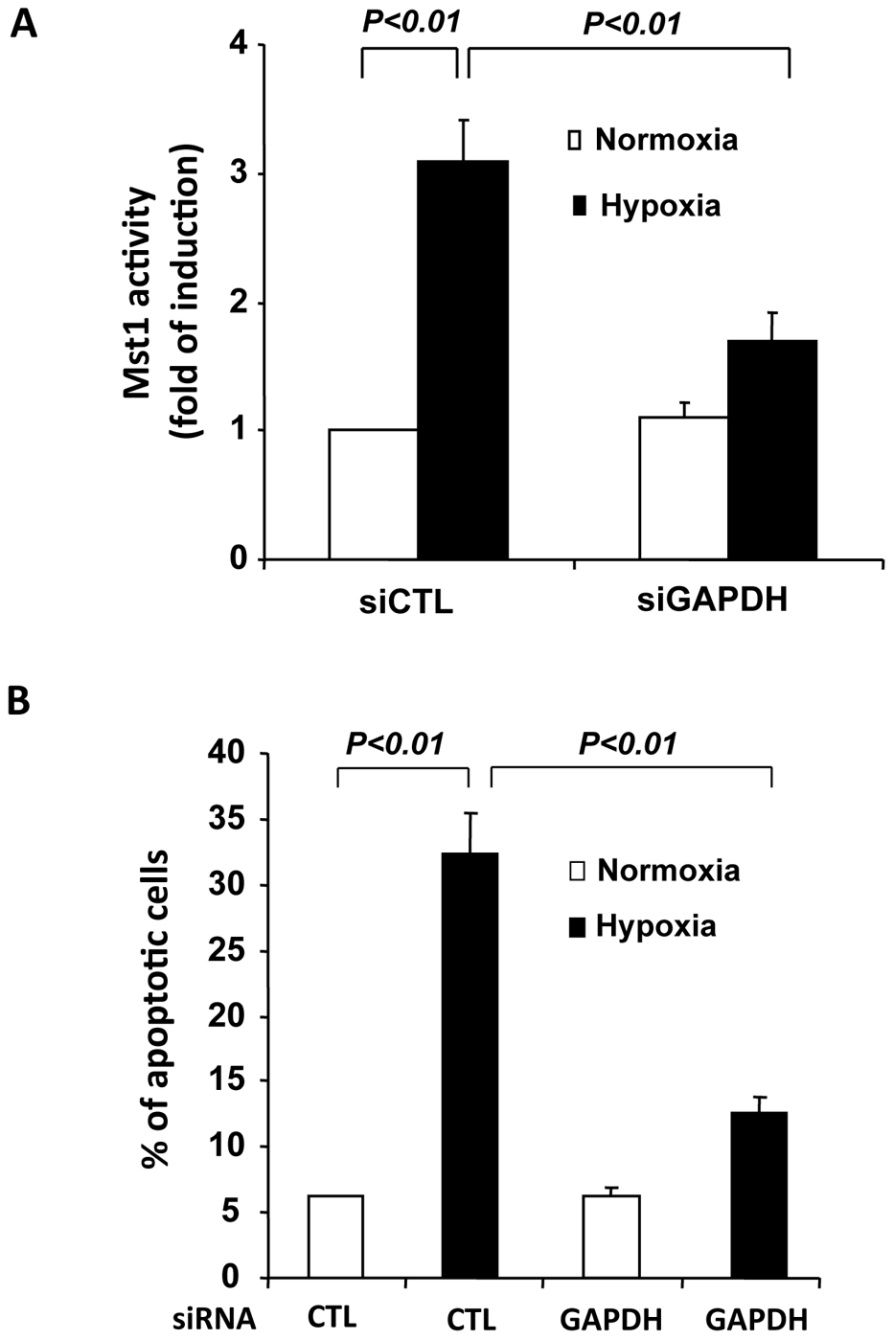
Knockdown of GAPDH attenuates Mst1 activation and cell apoptosis in response to hypoxia/reoxygenation. **A**, NRVMs were transfected with either control siRNA or GAPDH siRNA. 72 hours after transfection, cells were treated with hypoxia for 12 hours and reoxygenation for 24 hours. Mst1 was then immunoprecipitated and its activity was determined by an in vitro kinase assay using histone H2B as a substrate. **B**, NRVMs were transfected with either control siRNA or GAPDH siRNA. 72 hours after transfection, cells were treated with hypoxia/reoxygenation and the cell apoptosis was determined by using the TUNEL staining kit (Roche). Values are means ± SEM obtained from 4 experiments.

To further investigate the role of GAPDH in the regulation of Mst1 mediated cardiomyocytes, we used siRNA to knockdown the expression of GAPDH. Transfection of GAPDH siRNA (siGAPDH) reduced GAPDH expression by ∼80% in cardiomyocytes, as determined by both qRT-PCR (Figure 8A) and western blot analysis (Figure 8B). Knockdown of GAPDH expression was found to markedly attenuate Mst1 activity in response to chelerythrine treatment, as compared with that in cells transfected with control siRNA (siCTL) (Figure 8C). Moreover, knockdown of GAPDH expression markedly inhibited the cardiomyocyte apoptosis in response to chelerythrine, as determined by TUNEL staining (Figure 8D). However, transduction of cardiomyocytes with Ad-Mst1 (MOI = 50) fully restored the cardiomyocyte apoptosis induced by chelerythrine stimulation in cardiomyocytes transfected with GAPDH siRNA (Figure 8D). Importantly, knockdown of GAPDH also markedly inhibited hypoxia/reoxygenation induced Mst1 activation (Figure 9A) and cardiomyocyte apoptosis (Figure 9B). Collectively, these findings further indicate that GAPDH is a positive regulator of Mst1 activation in cardiomyocyte apoptosis.

## Discussion

Using the yeast 2-hybrid system and Mst1 as bait, we isolated several GAPDH cDNAs from a human heart cDNA library. The interaction of GAPDH with wild-type Mst1 was further supported by coimmunoprecipitation studies showing that in both cotransfected HEK293T cells and cardiomyocytes, GAPDH specifically interacts with Mst1. The interaction between GAPDH and Mst1 requires the kinase domain of Mst1 and it seems that the C-terminal catalytic domain of GAPDH mediates the binding of Mst1 in GAPDH. Interestingly, GAPDH is phosphorylated by Mst1 to a comparable extent as its known substrate MBP, suggesting that GAPDH is a good substrate of Mst1 at least in vitro. Furthermore, the functional consequence of this interaction was demonstrated by the ability of GAPDH to enhance Mst1 activity and Mst1-mediated apoptotic effects in cardiomyocytes. Indeed, inhibition of GAPDH expression attenuates the Mst1 activation and the Mst1 induced cardiomyocyte apoptosis in response to chelerythrine. These results suggest that the regulation of Mst1 activity by GAPDH may be an important determinant of cell survival in the heart.

GAPDH is a key enzyme in the glycolytic pathway, which catalyzes the conversion of glyceraldehyde-3-phosphate (G3P) to 1,3-biphosphoglycerate in the presence of NAD+ and inorganic phosphate [28]. Recently, accumulating evidence suggests that in addition to its canonical role in the glycolytic pathway, GAPDH functions as a key component in the regulation of many fundamental cellular functions [28]. Particularly, its roles in the nucleus and in the regulation of cell apoptosis have attracted significant attention [30], [31]. Indeed, increased expression and nuclear translocation of GAPDH has been implicated in the cell apoptosis in several cell types [30], [32], [33]. However, at this time, the molecular mechanism underlying the cell apoptosis triggered by GAPDH nuclear translocation remains elusive. Recently, nitrosative stress conditions have been shown to induce nitrosylation of GAPDH and its interaction with the ubiquitin ligase Siah1, which translocates GAPDH to the nucleus [30]. In the nucleus, GAPDH stabilizes the rapidly turning Siah1 complex, leading to ubiquitination and subsequent degradation of selected target proteins, thereby affecting apoptosis [30]. In addition, GAPDH has been shown to facilitate apoptosis when localized to mitochondria, where it induces the pro-apoptotic mitochondrial membrane permeabilization and the release of pro-apoptotic cytochome c [34]. The functional role of GAPDH in cardiomyocyte apoptosis has begun to be explored. For instance, the increased GAPDH gylcolytic activity induced by phenylephrine treatment has recently been shown to protect the cardiomyocytes from the starvation induced apoptosis [35] and this appears to disagree with the conclusion of the present study. The cause of this discrepancy may be due to the different external stimuli used for inducing apoptosis, since increased GAPDH activity can generate more ATP, which can counteract the decreased ATP levels during the starvation induced apoptosis. Interestingly, a recent report demonstrated that during nitric oxide (NO)-induced cardiomyocyte apoptosis, GAPDH is translocated to the nucleus of cardiomyocytes and overexpression of glutaredoxin protects cardiomyocytes against NO induced apoptosis through suppressing the translocation of GAPDH [36]. In the present study, our data demonstrated that GAPDH specifically binds to Mst1 and translocates to the nucleus during cardiomyocyte apoptosis. Inhibition of GAPDH attenuates Mst1 activation and Mst1 mediated cardiomyocyte apoptosis. Thus, our findings identified Mst1 as a novel downstream target of GAPDH in cardiomyocyte apoptosis.

The Mst1 signaling pathway plays an essential role in cell apoptosis [2]. Intact Mst1 is localized predominantly in the cytoplasm, however, in response to a variety of apoptotic stimuli, Mst1 is cleaved by caspases to produce the N-terminal constitutively active kinase domain and this cleavage markedly increases Mst1 kinase activity and translocates the cleaved Mst1 to the nucleus [4]–[6]. In the present study, our results demonstrated that both GAPDH and Mst1 translocated to the nucleus and strongly colocalized in the nucleus in response to chelerythrine. The interaction of GAPDH with Mst1 in the nucleus could be critical for the execution of cell apoptosis, since GAPDH can further augments Mst1 activity in the nucleus, thereby potentiating the Mst1 induced apoptosis. In addition, the interaction of Mst1 with GAPDH could further lead to GAPDH phosphorylation, therefore affecting its interaction with other downstream targets such as Siah1 and p300 in the nucleus [30], [31], thereby influencing cell apoptosis. Although the function of the Mst1 mediated GAPDH phosphorylation remains unknown, it would be very interesting to investigate whether Mst1 mediated GAPDH phosphorylation affects its other functions in the nucleus, such as mediating RNA nuclear export, gene transcription, mRNA stability, and telomere protection [37], [38].

The mechanism by which GAPDH activates Mst1 is not known, but certainly involves other components in Mst1 immunocomplexes, as such an increased Mst1 activity by GAPDH was only observed with immunoprecipitated Mst1 from HEK293 cells transfected with Mst1 cDNA. Indeed, the Mst1 kinase signaling cascade is tightly regulated by protein–protein interactions. Several proteins, including Rassf1, hWW45 [17], [19], PHLPP1 [20], and Death-associated Protein 4 (DAP4) [5], have been shown to interact with Mst1, thereby influencing the activities of MST1/2 kinases in mammalian cells. In this regard, it is attempting to speculate that the interaction of GAPDH with Mst1 may cause a conformational change of Mst1, thus affecting the formation of the Mst1/Hippo signaling complex with other proteins, such as Rassf1, hWW45, and Lats, which have recently been shown to play essential roles in the regulation of cardiomyocyte apoptosis and heart failure [39], [40]. These studies are ongoing.

In summary, we have found that GAPDH interacts with Mst1 kinase in cardiomyocytes. This interaction is specific and leads to a functional increase in Mst1 activity and Mst1 mediated cardiomyocyte apoptosis. These findings suggest that the GAPDH/Mst1 pathway may be an important therapeutic target for inhibiting myocardial apoptosis in ischemia-reperfusion injury and heart failure.
